# Long-term mechanical assisted circulation devices

**DOI:** 10.1590/1516-3180.2022.140303122021

**Published:** 2022-05-05

**Affiliations:** I MD, PhD. Attending Physician, Division of Cardiology, Instituto do Coracao, Hospital das Clinicas HCFMUSP, Faculdade de Medicina, Universidade de Sao Paulo, Sao Paulo, SP, BR.; II MD, PhD. Full Professor, Thoracic Surgery Program, Instituto do Coracao, Hospital das Clinicas HCFMUSP, Faculdade de Medicina, Universidade de Sao Paulo, Sao Paulo, SP, BR. Director, Scientific Department, Associação Paulista de Medicina, São Paulo, SP, BR.

Heart failure syndrome is the final presentation of a series of cardiac diseases. According to the Department of Informatics of the Brazilian National Health System (DATASUS), cardiovascular diseases are the third largest cause of hospitalizations in Brazil and heart failure is the main cause of cardiovascular hospital admissions in this country.

Despite all the advances in treatments for heart failure, a significant proportion of such patients evolve to states of refractoriness to clinical treatment. In these situations, it becomes necessary to use advanced procedures such as heart transplantation and long-term mechanical assisted circulation devices.

Heart transplantation is still the gold-standard treatment for refractory heart failure and the mean survival after this procedure is 11 years.^1^ However, a large proportion of such patients cannot benefit from this procedure either because of contraindications or because of lack of availability of organs.

In the light of this scenario, there has been great pressure to develop therapies that provide an alternative to transplantation. This has culminated in the introduction of long-term mechanical assisted circulation devices.^2^

The beginnings of the development of mechanical assisted circulation devices date back to 1950, when an extracorporeal circulation machine was first used. In 1964, the National Heart and Lung Institute of the United States created an artificial heart program. Twenty years later, in the 1980s, tests on the HeartMate XVE implant began.

HeartMate XVE was the first generation of long-term mechanical assisted circulation devices. It propelled the blood flow by means of a pulsatile pump that attempted to mimic the flow in the left ventricle. This device was tested in the Randomized Evaluation of Mechanical Assistance for the Treatment of Congestive Heart Failure (REMATCH) trial^3^ and was shown to provide a reduction of 48% in the risk of death over a one-year period. However, use of this device presented a high rate of complications such as thromboembolic events and hemolysis.

In 2007, a study comparing HeartMate XVE and HeartMate II, a second-generation device, was published. This new device had a different mechanism, consisting of continuous axial flow. Its technology brought in the advantage of being a smaller device that could be used by individuals of lesser build, with the expectation that it would be possible to use it for a long period, given that it only had a single moving part, its rotor.^4^

Subsequently, axial flow devices dominated in the field of long-term mechanical assisted circulation, for a long period. Centrifugal flow devices then emerged, which brought in the advantages of miniaturization, intrapericardial placement and a bearing-free rotor, achieved through the technologies of electromagnetic or hydrodynamic levitation. These devices reduced hemolysis and adverse events relating to hemocompatibility.^5–7^

The latest report from the Interagency Registry for Mechanically Assisted Circulatory Support (INTERMACS) showed that 77.7% of the long-term devices used in 2019 made use of magnetic levitation, thus showing the growth in this technology over recent years.^8^

Implantation of long-term mechanical assisted circulation devices can be indicated through the following strategies: 1 – As a bridge to candidacy: in situations of clinical conditions that prohibit heart transplantation but which if modifiable would allow the patient to become a candidate for transplantation (for example: pulmonary hypertension and neoplasias that are potentially curable); 2 – As a bridge to transplantation: in situations in which the device might provide hemodynamic support and clinical stability until the heart transplantation is performed, within a context of progressively increasing severity of the patient's condition and unavailability of an organ for transplantation within the short term; 3 – As destination therapy: in situations in which the device might provide hemodynamic support and clinical stability for a patient with refractory heart failure who presents contraindications for heart transplantation, thus enabling greater survival and better quality of life, in comparison with clinical treatment using medications.^9^

In Brazil, experience with these devices remains sparse,^10^ given their high cost and the potential growth in the number of heart transplantations that has occurred over recent decades.^11^ These factors have left Brazil well behind with regard current treatments for advanced heart failure.

Within the worldwide scenario, what we see is that improvements in the technology of long-term mechanical assisted circulation devices and in the expertise of medical teams have consequentially increased the survival of such patients. This has given rise to changes in the range of options for treating refractory heart failure. Long-term mechanical assisted circulation devices are increasing gaining space: not only at the transplantation point but also, especially, as destination therapy, which accounted for more than 70% of the indications in 2019.^8^
